# Highly Sensitive Electrochemical Sensor for Sunset Yellow Based on Electrochemically Activated Glassy Carbon Electrode

**DOI:** 10.3390/molecules27165221

**Published:** 2022-08-16

**Authors:** Yan Lu, Chengqi Bao, Jin Zou, Jinli Xiao, Wei Zhong, Yansha Gao

**Affiliations:** Key Laboratory of Crop Physiology, Ecology and Genetic Breeding, Ministry of Education, Key Laboratory of Chemical Utilization of Plant Resources of Nanchang, College of Chemistry and Materials, Jiangxi Agricultural University, Nanchang 330045, China

**Keywords:** activated glassy carbon electrode, electrochemical sensor, sunset yellow, electrochemical activation

## Abstract

Electrochemically activated glassy carbon electrode (AGCE) was fabricated and applied for sensitive and selective detection of sunset yellow (SY). The electroanalysis of SY was investigated by square wave voltammetry (SWV). Owed to the specific oxygen-contained functional groups and the outstanding conductivity of AGCE, the proposed sensor exhibits an enhanced oxidation peak current of SY when compared with non-activated glass carbon electrode (GCE). Under the optimal analytical conditions, the oxidation peak current is linear with SY concentration in the range of 0.005–1.0 μM. The low limit of detection is 0.00167 μM (S/N = 3). This method is applied for the detection of SY in the actual samples. The recovery is between 96.19 and 103.47%, indicating that AGCE is suitable for the determination of SY in beverage sample.

## 1. Introduction

Nowadays, food dyes are still an important issue in food industries. To make appealing colored products, manufacturers often add food pigments into food during processing [1,2]. As one of the most typical synthetic dyes, sunset yellow (SY) has been widely applied in the preparation of foods, due to its low cost, uniformity in color, and high stability [2,3]. The maximum daily intake of SY is strictly limited to 2.5 mg/kg according to the WHO health guidelines [4]. Excessive consumption can lead to a host of serious health problems, such as eczema, hepatocellular damage, and asthma [5,6,7]. Accordingly, the key to ensuring food safety is the establishment of an assay capable of being easy and rapid. Considering the food quality and safety, it is urgent to develop a convenient and effective method for the quick detection of SY.

To date, many techniques have been developed for quantitative SY analysis, including high-performance liquid chromatography (HPLC) [8,9], fluorescence [10], Raman [11], UV-visible absorption [12], and diffuse reflection FTIR spectroscopy [13]. Although these methods can offer good sensitivity, they still show some deficiencies, such as being time-consuming, having cumbersome preprocessing, and using expensive instrumentation [14,15]. Compared with the above methods, further attention has been paid towards the electrochemical method due to its unique advantages of quick response, simplicity, and low cost.

Since the reaction kinetics of bare electrode surfaces is slow, it could not satisfy the need of trace level sensing. So far, most of the electrochemical methods for SY have been focused on further improving the surface kinetics by using advanced materials modified on the surface of electrodes, such as poly(L-cysteine) [16], ZnO/cysteic acid nanocomposite [4], and Zinc oxide nanoflower [5]. However, the successful synthesis of materials is a complicated process with time-consuming steps, high cost, and strict reaction conditions [17]. In fact, direct oxidation of glass carbon electrode is an effective method to obtain promising sensing electrode due to the advantages of it being low cost, time-efficient, and easy to operate [18]. The oxidation treatment could produce abundant oxygen-containing functional groups, and new active sites of a high density of electronic states on the electrode surface [19], thus enhancing the electron transfer rate and enabling the strong adsorption to the analytes [20]. Until now, electrochemical sensors based on activated glass carbon electrode (AGCE) have been successfully achieved the sensitive detection of several substances, such as imidacloprid (IDP) [21], Uric Acid [22], and acetaminophen [23]. However, there is no study on the use of AGCE for the detection of SY.

**Scheme 1:**
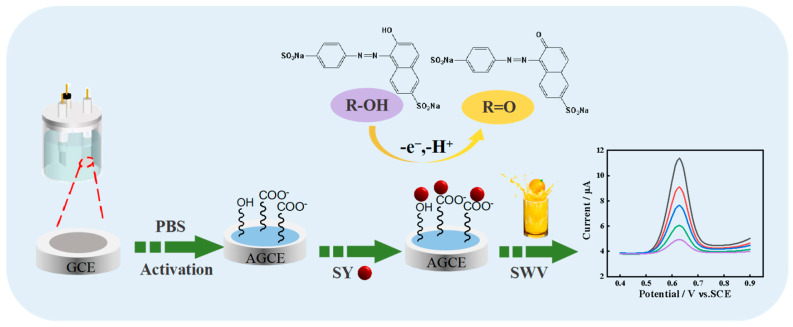
Activation steps of GCE and the redox mechanism of the SY at the AGCE.

Herein, a rapid and simple AGCE-based sensing platform for the sensitive determination of SY was fabricated. Electrochemical activation of the GCE was performed using the cyclic voltammetry method. The activation steps of GCE and the redox mechanism of the SY at the AGCE are shown in Scheme 1. Due to the excellent conductivity and abundant oxygen-containing functional groups of AGCE, the AGCE shows excellent electrocatalytic activity toward SY with a wide detection range and low detection limit. Moreover, the presented electrochemical sensor is successfully applied for SY detection in the actual samples.

## 2. Results and Discussions

### 2.1. Characterization of the AGCE

**Figure 1:**
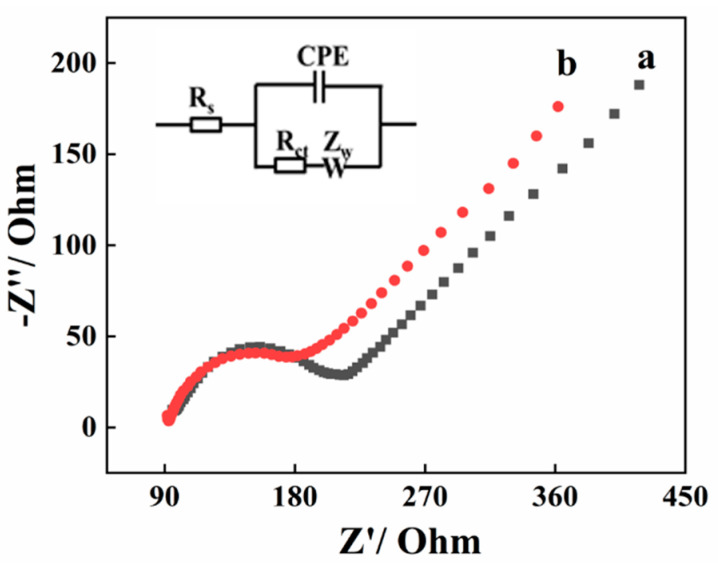
Nyquist plots of bare GCE (a) and AGCE (b) in 0.1 mM [Fe(CN)6]^3−/4−^ solution containing 1 M KCl. Inset: the equivalent circuit.

The conductive capacity of bare GCE and AGCE was investigated by the Electrochemical impedance spectroscopy (EIS). According to the reported studies, the diameter of the semicircle in the impedance spectrum corresponded to the charge transfer resistance (R_ct_) [24]. Figure 1 displays the Nyquist plot of the bare GCE (a) and AGCE (b), along with the equivalent electric circuit. In the equivalent circuit, R_S_ represents the electrolyte resistance, Z_W_ represents Warburg impedance, as well as R_ct_ represents charge transfer resistance. In comparison with bare GCE (135 Ω), the R_ct_ value of AGCE (95 Ω) decreases ascribing to the excellent conducting properties of AGCE.

### 2.2. Electrochemical Behavior of SY on Different Electrodes

**Figure 2:**
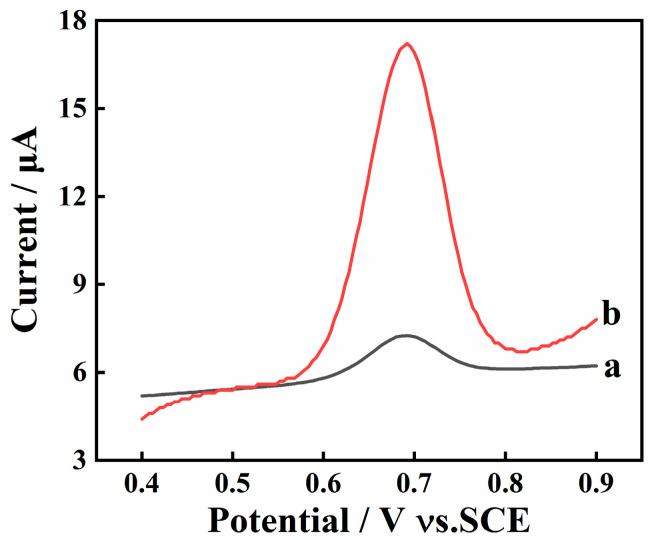
SWV curves of 10 μM SY in 0.1M PBS (pH 7.0) on bare GCE (a) and AGCE (b); Accumulation potential: 0 mV; Accumulation time: 15 s.

The electrochemical behavior of SY at bare GCE and AGCE was initially investigated. As shown in Figure 2, an obvious oxidation peak is observed around 0.68 V at GCE and AGCE. Furthermore, the peak current at AGCE is almost ten times higher than that at GCE. This is because the oxygen-containing functional groups generated after the activation step can efficiently enrich SY on the AGCE surface and amplify the detection signal.

### 2.3. Effect of Electrochemical Detection Technique

**Figure 3:**
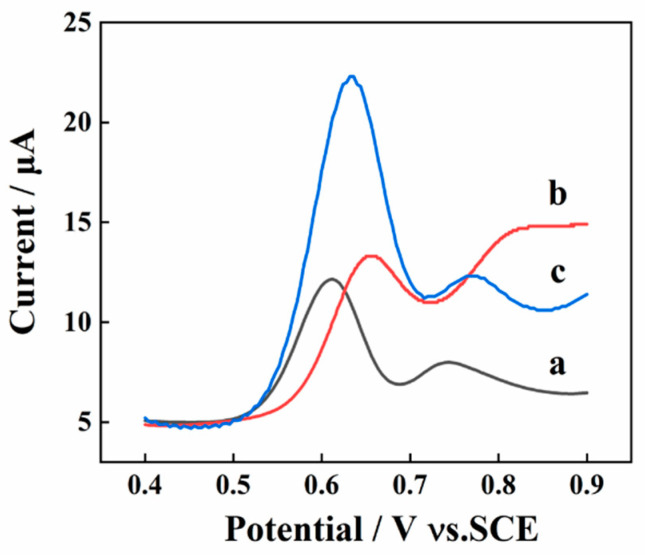
The electrochemical behavior of 10 μM SY on AGCEs by using DPV (a); step potential: 4.0 mV; pulse height: 0.05 s; pulse period: 0.5 s, LSV (b), and SWV (c) with a frequency of 15 Hz.

Different detection techniques (LSV, DPV, and SWV) were conducted for the collection of response signal of SY (10 μM) to gain the highest response signal of SY. As shown in Figure 3, the peak current obtained from the SWV method is much higher than the other two. As a result, the SWV is utilized as the detecting technique for the determination of SY.

### 2.4. Optimization of the Experimental Conditions

#### 2.4.1. Effects of Accumulation Time

**Figure 4:**
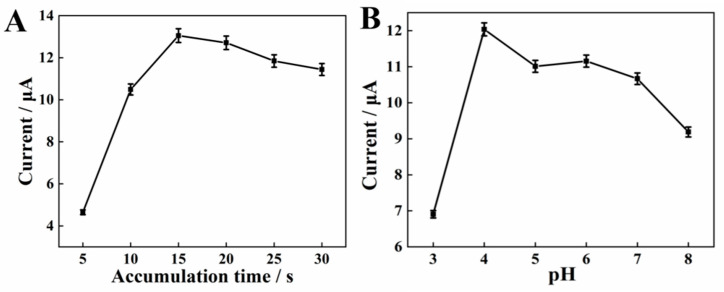
Effects of accumulation time (**A**), activation pH value (**B**) on the anodic peak currents of 10 μM SY.

The influence of accumulation time on the oxidation response of SY was examined at the AGCE. The anode peak current improves significantly as shown in Figure 4A, when the accumulation time increases from 5 s to 15 s with an accumulation potential of 0 mV. When further increasing the accumulation time to 30 s, the current response is gradually reduced, indicating that the adsorption of SY on the AGCE surface reaches a saturation state. Accordingly, 15 s is considered as the optimal accumulation time for the assay.

#### 2.4.2. Effects of Electrochemical Activation pH

**Figure 5:**
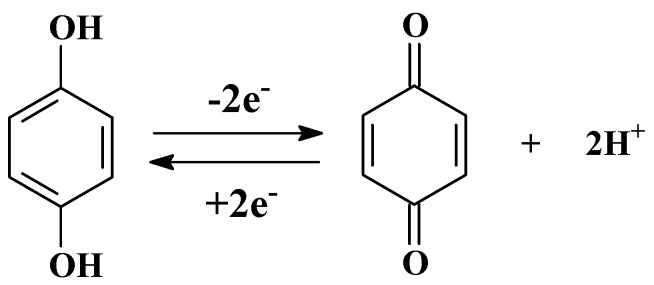
The redox reaction of quinone.

A different activation pH could produce different effects, including obtaining the various amounts of oxygen-containing functional groups. Consequently, the effects of different activation pH were explored before the detection of SY. The polished GCE was activated in PBS at various activation pH (3.0–8.0) and the peak oxidation current of SY on the AGCE was subsequently recorded. Figure 4B shows the SWV curves of SY, the peak current of SY increases with increasing pH from 3.0 to 4.0, and then gradually decreases with pH rises to 8.0. Generally speaking, the peak current in the cyclic voltammogram of a carbon electrode is related to the redox reaction of quinone (Figure 5) [25,26]. At lower pH values, too much H^+^ in solution could restrain the redox reaction, thus reducing the number of oxygen-containing groups on the electrode surface. Hence, pH = 4.0 is selected as the optimal pH for the activation electrode.

#### 2.4.3. Effects of pH

**Figure 6:**
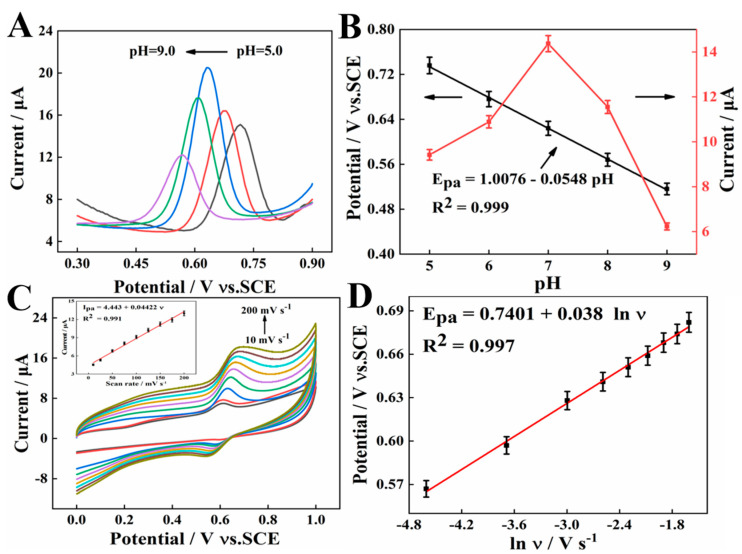
(**A**) SWV curves of 10 μM SY at AGCE in PBS with different pH. (**B**) The plots of pH vs. E_pa_. (**C**) CVs at the pH 7.0 PBS with different scan rates (from 10 to 200 mV s^−1^) (Inset The relationship of I_pa_ and ν). (**D**) The linear relationship between the peak potential and lnν.

The effects of pH on the oxidation peak current of 10 μM SY were explored by SWV in the pH ranges from 5.0 to 9.0. As seen in Figure 6A, the oxidation peak current of SY gradually increases with the pH value changing from 5.0 to 7.0, and then decreases with the increase in pH from pH 7.0 to 9.0. Therefore, pH = 7.0 is selected as the optimal pH for the following studies. In addition, the anodic peak potential shifts toward more negative potentials with pH increasing from 5.0 to 9.0 (Figure 6B). The linear relationship between anodic peak potential and pH can be expressed as: E_pa_ (V) = 1.0076 − 0.0548 pH (R^2^ = 0.999), where the slope (−0.0548) of the equation is quite close to the theoretical value of −0.059 mV/pH, indicating that the same number of transferred proton and electron [27].

#### 2.4.4. Effects of Scan Rate

The influence of the scan rate on the oxidation current response of 10 μM SY was studied in the range of 10–200 mV s^−1^ in Figure 6C. It could be shown that the peak current response increases linearly at the increment of scan rate, along with the linear equation of I_pa_ (μA) = 4.443 + 0.0442 ν (mV s^−1^) (R^2^ = 0.991). It was indicated that the electro-oxidation of SY at AGCE is an adsorption-controlled process. In addition, the relationship between the peak potential (E_p_) and the logarithm of scanning rate (lnν) was assessed. As seen in Figure 6D, the peak potential is linearly proportional to the Napierian logarithm of scan rate (lnν), corresponding to the linear equation, E_pa_ = 0.7401 + 0.038 lnν (R^2^ = 0.997). Based on Laviron’s theory, $E_{\mathrm{pa}}=E^{0}-\frac{\mathrm{RT}}{\alpha \mathrm{nF}}+\frac{\mathrm{RT}}{\alpha \mathrm{nF}}\ln\nu$, where α and n refer to the electron transfer coefficient, and the number of the electron transfer, respectively [28]. The slope for the linear region is equal to RT/αnF. According to the slope values, the αn value was computed as 0.67. Since α is 0.55 for irreversible electrochemical reactions, n is determined to be 1, which suggests that a proton and an electron are involved in SY oxidation.

### 2.5. Electrochemical Detection of SY

**Figure 7:**
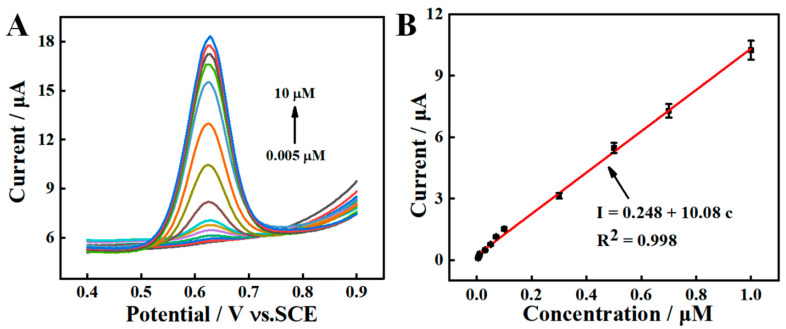
(**A**) SWVs response for various concentrations of SY at AGCE, (**B**) Calibration curve of SY from SWV.

Under the optimal experimental conditions, different concentrations of SY are quantified on AGCE in PBS (pH = 7.0). As displayed in Figure 7A, the oxidation peak current demonstrates a rising trend over a concentration range of 0.005–10.0 μM. As shown in Figure 7B, a linear curve is plotted at a concentrations range of 0.005–1.0 μM. The linear regression equations could be described as I (μA) = 0.248 + 10.08 c (μM) (R^2^ = 0.998). The limit of detection (LOD) is calculated to be 0.00167 μM (S/N = 3). Compared with the reported literatures (Table 1), the AGCE sensor exhibits a low LOD and wide linear range.

### 2.6. Repeatability, Reproducibility, and Anti-Interference

**Figure 8:**
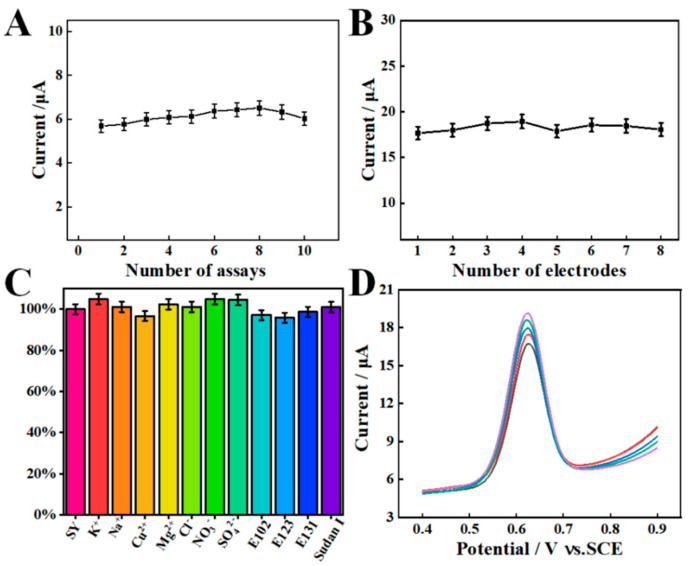
(**A**) Repeatability and (**B**) reproducibility of the AGCE sensor towards 10.0 μM SY. (**C**) Anti-interference of the AGCE for the detection of 10.0 μM SY. (**D**) SWV response of SY in the beverage samples in a 1.0 M PBS (pH 7.0).

The repeatability of one AGCE was investigated by ten successive detections of 10.0 μM SY under optimized experimental conditions (Figure 8A). The relative standard deviation (RSD) is 4.51%, suggesting that the AGCE has a satisfying repeatability. The reproducibility was examined by using eight independent AGCEs, and the RSD is calculated to be 2.45% (Figure 8B). This result indicates that AGCE possesses excellent reproducibility.

The anti-interference of the proposed AGCE sensor was evaluated by measuring the SWV responses of 10.0 μM SY with various interference matrices. Specifically, 100-fold concentration of KNO_3_, NaCl, CuSO_4_, and MgCl_2_ as well as 10 μM tartrazine (E102), amaranth (E123), patent blue (E131), and Sudan I were used as interference studies. As shown in Figure 8C, the presence of interferences has no significant effect on the electrochemical determination of SY. The above result demonstrates that the AGCE sensor possesses excellent anti-interference ability for SY detection.

### 2.7. Practical Application

The practical applicability of the AGCE sensor was studied via detecting the SY in a beverage sample. The beverage sample was purchased from the local market. Before the measurement, the beverage sample was filtered through a 0.45 μm membrane to remove the impurities, then diluted by 100-fold with 0.1 M PBS with pH 7.0 and spiked with different concentrations. After that, the current response was detected using the SWV method under the best conditions (Figure 8D). As shown in Table 2, the recoveries range from 96.19% to 103.47% with the RSD values from 2.83% to 4.46%. All these results prove that the proposed sensor has a good feasibility for the detection of SY in actual samples.

## 3. Experimental

### 3.1. Reagents and Apparatus

Sunset yellow, lemon yellow, Sudan I, amaranth, patent blue, potassium ferricyanide, and potassium ferrocyanide were obtained from Aladdin Reagent Co., Ltd (Shanghai, China). Phosphate buffer solution (PBS) was prepared by mixing 0.1 mM potassium dihydrogen phosphate and 0.1 mM dipotassium phosphate hydrogen phosphate. All chemicals were of analytical grade and were used directly.

All electrochemical experiments were performed with a CHI 760E Electrochemical workstation (Chenhua Instrument Co. Ltd., Shanghai, China). There is a conventional three-electrode system in use with an activated or bare glassy carbon electrode (GCE) as working electrode, a saturated calomel electrode (SCE) as the reference electrode, and a platinum (Pt) wire as the counter electrode.

### 3.2. Preparation of AGCE

Before being used, the GCE with a diameter of 3.0 mm was thoroughly polished with alumina powder to a mirror-like shape, followed by an ultrasonic cleaning in deionized water for 1 min. Unless otherwise stated, a freshly prepared GCE was activated in 0.1M PBS at the scan rate of 100 mV s^−1^ by cycling in the potential range between −2.0 V and 2.0 V vs. SCE for 20 segments (10 cycles), in order to achieve the AGCE.

### 3.3. Electrochemical Method

The supporting electrolyte for the determination of SY was in use of a 0.1M PBS with a pH of 7.0. After an accumulation time of 15 s, the square wave voltammograms (SWVs) were performed at the potential range of 0 to 1.0 V. Experimental parameters of the SWV were set with a step potential of 4.0 mV, an accumulation potential of 0 mV, a frequency of 15 Hz, and an amplitude of 25 mV.

## 4. Conclusions

In this paper, a sensitive electrochemical sensor based on AGCE was established for the detection of SY. The rich oxygen-containing functional groups and outstanding conductivity of AGCE increases the electrochemical performance. Under the optimized conditions, the proposed sensor showed an excellent catalytic activity towards SY with a relatively lower LOD of 0.00167 μM. Additionally, the AGCE sensor shows excellent selectivity, and reproducibility towards the detection of SY. More importantly, the AGCE sensor is successfully applied to the detection of SY in beverage samples with desirable recoveries of 96.19–103.47%. Therefore, the fabricated AGCE sensor has broad application prospects for monitoring SY in practical applications.

## Figures and Tables

**Table 1:** Comparison with other reported electrochemical sensors for SY detection.

Electrode Material	Linear Range (μM)	LOD (μM)	Reference
ZnO-cysteic acid/GCE	0.1–3.0	0.030	[4]
GO ^a^-MWCNT ^b^/GCE	0.09–8.0	0.025	[29]
poly(L-cysteine)/GCE	0.008–0.7	0.004	[16]
GN ^c^-Ni/GCE	0.0074–0.442	0.0022	[30]
rGO ^d^/Au-Pd/GCE	0.331–0.686	0.0015	[31]
AGCE	0.005–1.0	0.00167	This work

Note: ^a^: graphene oxide; ^b^: multi-wall carbon nanotube; ^c^: graphene; ^d^: reduced graphene oxide.

**Table 2:** Determination of SY in the beverage samples (*n* = 5).

Samples	Added (μM)	Found (μM)	Recovery (%)	RSD (%)
1	1	0.976	97.6	4.46
2	3	3.036	101.2	2.83
3	5	4.865	97.3	3.69
4	7	7.243	103.47	4.2
5	9	8.657	96.19	3.11

## Data Availability

The data presented in this study are available in article.

## Sample Availability

Samples of the compounds are not available from the authors.
